# Surgical Treatment for Severe Scoliosis in Patients with Reduced Cardiorespiratory Function after Surgery for Congenital Heart Disease: A Report of Two Cases

**DOI:** 10.1155/2018/4610796

**Published:** 2018-09-25

**Authors:** Hayato Kinoshita, Naohisa Miyakoshi, Michio Hongo, Akiko Misawa, Daisuke Kudo, Yoichi Shimada

**Affiliations:** ^1^Department of Orthopedic Surgery, Akita Kousei Medical Center, 1-1-1 Iijima-Nishibukuro, Akita 011-0948, Japan; ^2^Department of Orthopedic Surgery, Akita University Graduate School of Medicine, 1-1-1 Hondo, Akita 010-8543, Japan; ^3^Akita Prefectural Center on Development and Disability, 3-128 Kamikitate Hyakuzaki Suwanosawa, Akita 010-1407, Japan

## Abstract

**Purpose:**

Congenital heart disease (CHD) is associated with an increased risk of scoliosis. The prognosis of scoliosis patients with CHD has improved because of advances in cardiac care. As a result, the frequency of surgery for scoliosis in this population has increased, although the risk of perioperative complications remains high. We treated two patients with CHD who underwent surgery for severe scoliosis. To avoid perioperative complications, we evaluated the preoperative cardiac status and anesthetic risks before posterior correction and fixation in both patients.

**Methods:**

An expert anesthesiologist evaluated the anesthetic risk in each case, and an adequate reservoir of autologous blood was collected preoperatively. The patient in case 1 was at risk of significant blood loss and required extremely careful operative technique. The patient in case 2 had low cardiac output preoperatively. We therefore performed a thorough preoperative cardiac evaluation. Both patients were admitted to the intensive care unit postoperatively.

**Results:**

Neither patient suffered serious complications, and both achieved favorable outcomes.

**Conclusions:**

Appropriate surgical technique and teamwork among experts are the keys to success in patients with severe scoliosis and CHD.

## 1. Introduction

Patients with congenital heart disease (CHD) are at increased risk of developing scoliosis. Van Biezen et al. reported that all patients who underwent left thoracotomy developed secondary scoliosis [1]. The exact mechanisms of scoliosis development, however, are unclear. In contrast, Reckles et al. reported that the incidence of scoliosis did not increase after surgery for CHD [2]. In either case, the prognosis of CHD patients has improved because of advances in cardiac care, and the frequency of surgery for scoliosis in this population increased as a result. When patients with a history of previous open-heart surgery also have decreased cardiac or pulmonary function, surgical treatment for scoliosis carries a relatively higher risk of inducing cardiopulmonary events. We present the cases of two patients with scoliosis who had undergone previous surgery for CHD. We describe the procedures used to treat their scoliosis and the perioperative risks associated with these procedures.

## 2. Materials and Methods

Two patients who developed severe scoliosis after surgery for CHD were treated at our institution. We evaluated their spines according to three regions—proximal thoracic, main thoracic, and lumbar—with the apex of the proximal thoracic region between T1 and T5, the apex of the main thoracic region between T5 and T12, and the apex of the lumbar region between T12 and L5. We then evaluated cardiac status according to the three-group method described by Kadhim et al. [3], which is as follows: group S, patients with a functional single cardiac ventricle or whose cardiac anatomy necessitates single-ventricle palliative surgery; group 2N, patients with two ventricles who have no significant functional or anatomic cardiac abnormalities; and group 2R, patients with two ventricles with significant residual cardiac abnormalities.

The present patients' preoperative anesthetic risk was determined by an anesthesiologist according to the criteria outlined by the American Society of Anesthesiologists (ASA). Cyanosis was defined as oxygen saturation <94% at rest in room air. In both cases, pediatric cardiologists advised us to be aware of possible perioperative hemodynamic instability and, if present, to ensure continuous direct arterial blood pressure measurements and central venous catheterization for the administration of cardiovascular agonists, as needed, intraoperatively and postoperatively. Anesthesiologists performed this monitoring and minimized blood loss by using a cell saver intraoperatively to salvage any blood loss. In addition, pediatric cardiologists advised us to measure oxygen saturation regularly—not only intraoperatively and postoperatively but also preoperatively—to monitor for worsening heart function.

## 3. Case Reports

### 3.1. Case 1

**Figure 1:**
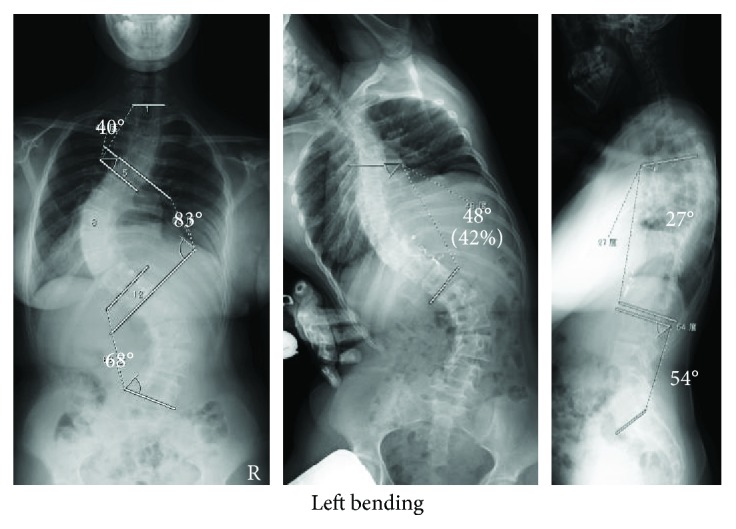
Case 1: preoperative radiographs (from left to right: anteroposterior, side-bending, lateral). Anteroposterior radiograph shows an 83° left convex main thoracic curve, a 40° proximal thoracic curve, and a 68° lumbar curve. Side-bending radiograph shows 42% flexibility at the main thoracic curve. Lateral radiograph shows thoracic kyphosis of 27° and lumbar lordosis of 54°.

A girl born with a right single ventricle had undergone bidirectional Glenn shunt and Fontan procedure during childhood. When she was 11 years old, she was diagnosed with scoliosis that gradually worsened until, at age 15, she was referred to our department. An anteroposterior radiograph showed an 83° left convex main thoracic curve, a 40° proximal thoracic curve, and a 68° lumbar curve. The C7–central sacral vertical line (CSVL) was measured as the horizontal distance between the C7 plumb line and the CSVL, which was drawn through the center of the sacrum perpendicular to the iliac crests. The patient's C7–CSVL distance was within 20 mm, indicating that coronal balance of the spine was maintained [4]. Thoracic kyphosis was 27°, and lumbar lordosis was 54° (Figure 1). Side-bending tests showed 42% flexibility at the main thoracic curve. The patient suffered from restrictive lung function (forced vital capacity 48.6% and forced expiratory volume in 1 s 56.7%), hypoxemia (pO_2_ 68.8 mmHg and pCO_2_ 35.8 mmHg), and chronic heart failure, which had resulted in a slightly elevated brain natriuretic peptide level (38.4 pg/dl) and aortic regurgitation. Her oxygen saturation was <94% at rest in room air. Based on these findings, the patient's preoperative cardiac status was defined as group S, her anesthetic risk was classified as ASA 2, and she met the criterion for cyanosis. A reservoir of 1600 ml of autologous blood was collected preoperatively.

Posterior release was performed with Ponte osteotomy at every level and dissection between the transverse process and the rib around the apex. Posterior fixation was performed with pedicle screws and 5.5 mm titanium composite metal rods. The rod-rotation technique was used for spinal correction. Operation time was 396 min, and intraoperative blood loss was 1155 ml. The total amount of preoperatively collected autologous blood was returned to her during a period starting intraoperatively and ending postoperatively.

**Figure 2:**
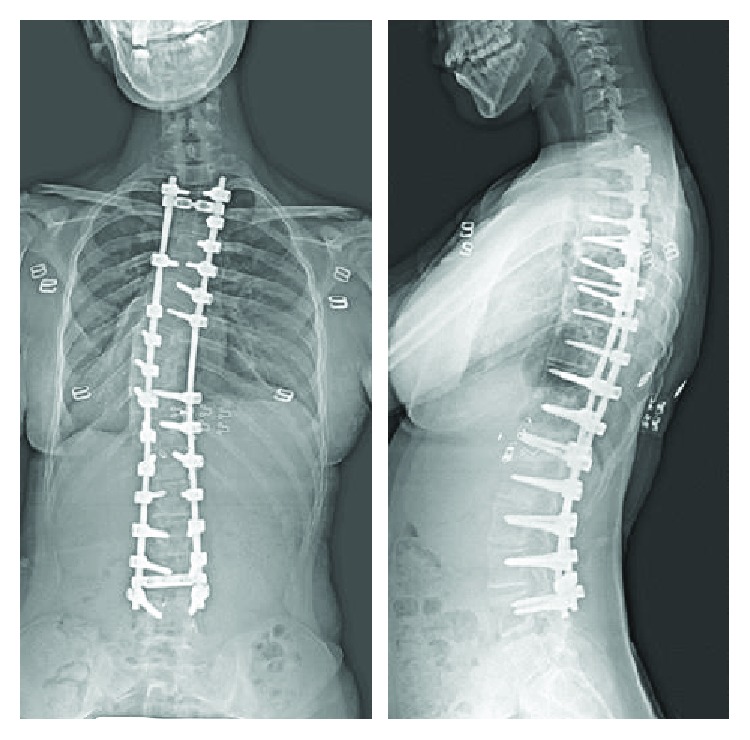
Case 1: postoperative radiographs (anteroposterior, lateral) 4 years after surgery show adequate curve correction and balance.

A postoperative radiograph showed 75% improvement in the patient's main thoracic curve. She remained in the intensive care unit for 2 days. On the first postoperative day, endotracheal intubation was maintained for respiration support, and the positive end-expiratory pressure was kept as low as possible to avoid deterioration of blood circulation caused by high pulmonary vascular resistance. On the second postoperative day, the patient's respiratory condition and blood pressure were stable, and she was extubated. There were no perioperative complications. At a 4-year follow-up visit, she was seen to have continued adequate curve correction and balance (Figure 2).

### 3.2. Case 2

A girl born with cyanosis and complete transposition of the great arteries had undergone an arterial switch operation, division of patent ductus arteriosus, and closure of patent foramen ovale soon after birth. At 3 years of age, she underwent surgery on her left coronary and pulmonary arteries. At age 5, she was diagnosed with scoliosis, which gradually worsened. The patient was referred to our department when she was 6 years old, and brace treatment was initiated. At 9 years of age, a left-sided abnormality of the abdominal skin reflex was noticed, and magnetic resonance imaging revealed an Arnold–Chiari malformation with syringomyelia, which was treated with suboccipital decompression.

**Figure 3:**
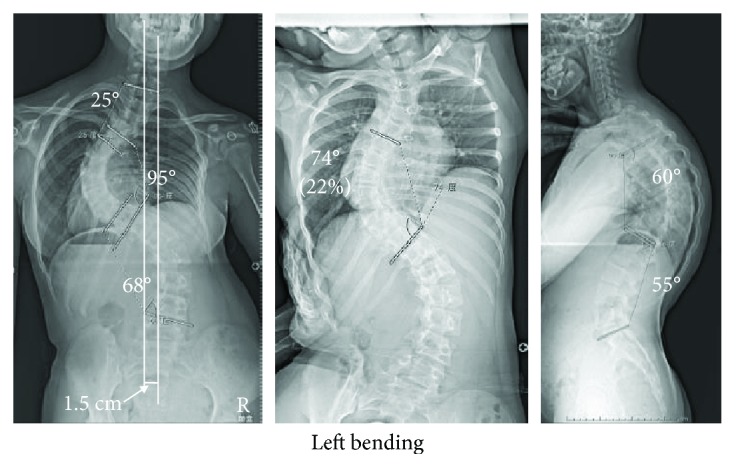
Case 2: preoperative radiographs (from left to right: anteroposterior, side-bending, lateral). Anteroposterior radiograph shows a major thoracic curve of 95° with a central sacral vertical line 1.5 cm to the left of the vertical plumb line. Side-bending radiograph shows 22% flexibility at the main thoracic curve. Lateral radiograph shows thoracic kyphosis of 60° and lumbar lordosis of 55°.

At age 10, the patient's scoliosis had progressed further, resulting in a major thoracic curve (95°) with a central sacral vertical line 1.5 cm to the left of the vertical plumb line (Figure 3). Side-bending test results showed 22% flexibility at the main thoracic curve. Prior to the latest operation, low cardiac output (left ventricular ejection fraction 45%–50%) and hypotension (85/53 mmHg) were present. Accordingly, the patient was considered to have group 2R preoperative cardiac status, and her anesthetic risk was classified as ASA 2. A reservoir of 2340 ml of autologous blood was collected preoperatively.

The same operative technique as was used in case 1 was performed for this patient but with different instruments. The operative time was 388 min, and intraoperative blood loss was 1806 ml. The entire amount of preoperatively collected autologous blood was returned to her starting intraoperatively and ending postoperatively. Postoperative radiography showed 74% improvement in the patient's main thoracic curve, and the thoracic kyphosis was corrected from 60° to 31°.

**Figure 4:**
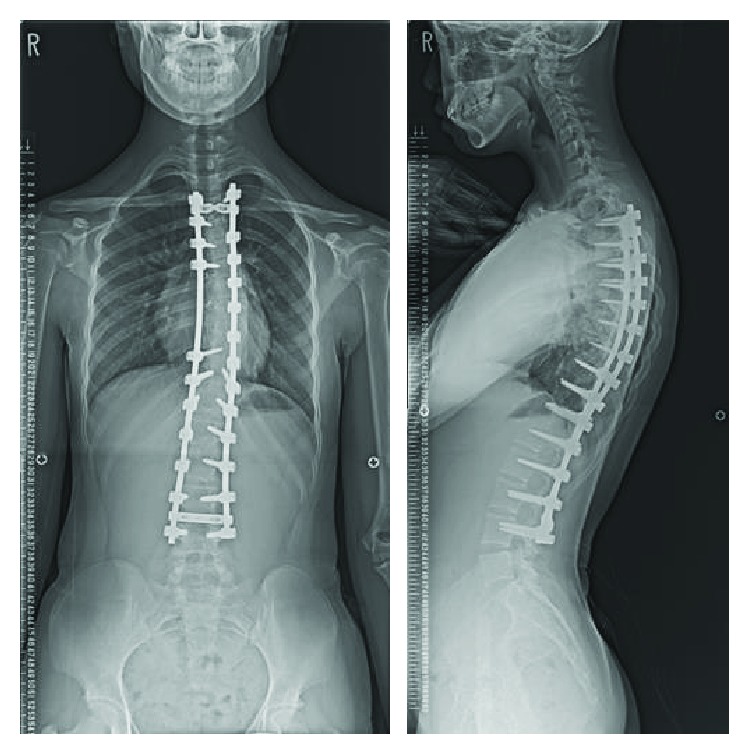
Case 2: radiographs (anteroposterior, lateral) 2 years after surgery show adequate curve correction and balance.

The patient remained in the intensive care unit for 3 days. On the first postoperative day, her systolic blood pressure fell temporarily to 50–60 mmHg. After semi-Fowler's positioning and a rapid intravenous drip infusion, her systolic blood pressure was maintained at around 100 mmHg. On the second day, the patient's blood pressure was stable. Her respiratory rate, tidal volume, and oxygenation were maintained unless her ventilation was switched from synchronized intermittent mandatory ventilation to spontaneous respiration, so she was extubated. After extubation, because the patient's oxygenation was somewhat poor, oxygen administration was continued until the third postoperative day. Thereafter, the patient experienced no perioperative complications. At the 2-year follow-up visit, she exhibited adequate curve correction and balance (Figure 4).

## 4. Discussion

Several studies have discussed the incidence of scoliosis after open-heart surgery [5, 6]. Kawakami et al. [5] reported that the incidence of scoliosis is higher among patients with CHD than in the general population (2%–19% vs. 2%–3%, respectively). These authors also reported that markedly fewer patients exhibited scoliosis before cardiac surgery than afterward (11 vs. 63 patients, respectively). Ruiz-Iban et al. [6] reported that many patients with CHD, especially those at age <18 months, develop scoliosis after undergoing procedures that included median sternotomy. These findings suggest that not only the Arnold–Chiari malformation but also the cardiac operation may have contributed to the development of scoliosis in the patient described in our case 2. In contrast, other studies have found that cardiac surgery does not increase the incidence of scoliosis [2, 7]. Further study is needed to confirm the risk factors for scoliosis.

Correcting severe scoliosis involves long surgical procedures and is associated with significant blood loss. Hence, intraoperative and postoperative complications are common, especially in patients with a history of CHD surgery. Hedequist et al. reported that patients with Fontan circulation (with poor systemic circulation and poor cardiac status) experienced a variety of complications after surgery for scoliosis, including paraparesis caused by vascular infarction affecting the midthoracic spinal cord and acute tubular necrosis [8]. Another study found that, during posterior spinal fusion for idiopathic scoliosis in patients with Fontan physiology, the venous pressure was markedly elevated because of being in the prone position, resulting in a prolonged prothrombin time and increased blood loss [9]. Evans et al. also suggested that patients with Fontan circulation have high venous pressure, particularly while in the prone position. Hence, operations requiring the prone position in these patients may result in significant blood loss [10]. Considering these concerns, we took extra care during the operation to avoid excessive blood loss in case 1.

In contrast, several researchers have reported a lower intraoperative cardiac index in patients undergoing surgery in the prone position [11, 12]. Brown et al. described changes in the cardiac indices of children undergoing scoliosis surgery in the prone position [13]. The authors used a Jackson frame in all cases, which reduced the risk of elevated venous pressure, but the patients' cardiac indices still fell. Therefore, the authors thought that increased intrathoracic pressure may be related to the lower cardiac index. Our patient in case 2 had low cardiac output, and therefore it was important to conduct an adequate preoperative cardiac evaluation.

Because surgery for scoliosis affects cardiopulmonary function, accurate preoperative assessment and appropriate intraoperative care are especially important for CHD patients undergoing scoliosis surgery. Perioperative care is also important for these patients. Particularly, excessive blood loss tends to occur in patients with Fontan circulation. Hypotensive anesthesia is often useful to prevent bleeding, although CHD patients with unstable hemodynamics can easily develop hypoperfusion. Evans et al. reported that operations during which the patient must remain prone for more than 4 h were associated with an increased risk of bleeding and hypotension, even when the patient was initially hemodynamically stable [10]. Patients with Fontan circulation (as in case 1) receive lifelong anticoagulant therapy, which makes the perioperative use of hemostatic agents difficult. Both operations described in this study involved long operation times. Because there was close collaboration between the pediatric cardiologist and anesthetist, adequate preoperative evaluation, and appropriate perioperative care, the patients had satisfactory outcomes.

The main limitation of our study is that it involved only two cases. Further studies are needed to determine definitively whether our approach reduces the risk of perioperative complications in CHD patients undergoing surgery for scoliosis.

## 5. Conclusion

The prognosis of CHD patients has improved, and many patients can now undergo surgery safely for their scoliosis. We performed scoliosis surgery in two patients with CHD and obtained satisfactory results, with no serious complications in either patient. Nevertheless, we remain vigilant and cautious as CHD patients sometimes exhibit unexpected hemodynamics and respiratory conditions. We believe that the combination of appropriate surgical technique and cooperative teamwork among experts is the key to success in such cases.
